# Quantitative Single‐Cell Comparison of Sensitization to Radiation and a Radiomimetic Drug for Diverse Gold Nanoparticle Coatings

**DOI:** 10.1002/smsc.202400053

**Published:** 2024-06-16

**Authors:** Douglas Howard, Tyron Turnbull, Puthenparampil Wilson, David John Paterson, Valentina Milanova, Benjamin Thierry, Ivan Kempson

**Affiliations:** ^1^ Future Industries Institute University of South Australia Mawson Lakes South Australia 5095 Australia; ^2^ Department of Nuclear Medicine University Hospital Essen Hufelandstrasse 55 45122 Essen Germany; ^3^ UniSA STEM University of South Australia Mawson Lakes South Australia 5095 Australia; ^4^ Department of Radiation Oncology Royal Adelaide Hospital Adelaide South Australia 5000 Australia; ^5^ Australian Synchrotron ANSTO 800 Blackburn Road Clayton Victoria 3168 Australia

**Keywords:** cancer, DNA damage, double‐strand breaks, nanomedicine, physical chemistry, radiotherapy, *γ*H2AX

## Abstract

Metal‐based nanoparticles (NPs) have entered clinical use for enhancing radiotherapy, but the underlying mechanisms remain ambiguous. Herein, single‐cell analysis of two cell lines in response to megavolt irradiation and a radiomimetic drug, neocarzinostatin (NCS) after coculture with gold NPs with different surface coatings, polyethylene glycol (AuPEG), PEG, and transferrin (AuT) or silica (AuSiO_2_), is reported. Different surface chemistry presents a major challenge for objective comparison between the biological impacts where major differences in cell‐uptake exist. AuSiO_2_ NPs are the most efficient for promoting radiosensitization despite being associated with cells 10 times less than the actively targeted AuT NPs. Conversely, for cells exposed to NCS, AuSiO_2_ NPs impede the radiomimetic action and promote cell survival. AuT NPs enhance death of cells in combination with NCS showing that NPs can sensitize against cytotoxic agents in addition to radiation. While NPs contribute to radiosensitization (or enhancing/impeding chemotherapeutic drug activity), due to cell and cell line heterogeneity, the ultimate radiosensitivity of a cell appears to be dominated by its inherent radiosensitivity and how this cell‐regulated response is manipulated by NPs. This is evidenced through comparison of radiobiological response of cells with equivalent NP association rather than equivalent coculture conditions.

## Graphical abstract

Nanoparticles (NPs) can sensitize cancer cells to both clinically relevant radiation energies and chemotherapy but the effects depend on cell and NP surface characteristics. When considering cells with identical quantities of internalized NPs, different surface chemistry results in different radiobiological effects. This shows that physical radiation dose deposition processes do not dominate radiosensitization and reveals new opportunities for sensitizing cancers.

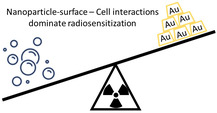

## 1 Introduction

In 2020, cancer was responsible for 10 million deaths worldwide^[^ ^1^ ^]^ and is expected to rise by 56% over the next 20 years,^[^ ^2^ ^]^ underpinning the need to understand and treat these diseases. The biological microenvironment is complex and high levels of heterogeneity between patients and within individual tumors results in treatment being unable to utilize a “one‐size‐fits‐all” model. Treatment includes combinations of chemotherapy, targeted therapies, immunotherapies, surgery, and/or radiation therapy, where radiation therapy is currently utilized in more than 50% of all cancer treatments in countries where infrastructure is available.^[^ ^1^, ^3^ ^]^ In this context, nanoparticle (NP) formulations have been developed to improve delivery of cytotoxic agents to tumors or enhance the effects of radiotherapy.

Therapeutic efficacy of X‐ray radiation therapy is dependent on the degree of tumor oxygenation as ionizing radiation (IR) generates reactive species such as hydroxyl radicals (•OH), superoxide (O2•−) and other reactive oxygen species (ROS), which cause DNA single‐ and double‐strand breaks.^[^ ^2^, ^4^ ^]^ This requires the prescription of increased radiation dose to overcome hypoxic desensitization of tumor; however, practically delivering higher doses is limited by increased probability of inducing unacceptable toxicities to normal tissues or secondary cancer induction.^[^ ^5^, ^6^ ^]^ Engineering advances have led to excellent conformation of radiation to treatment volumes, yet curative doses are still challenging to achieve for many cancers. This has motivated development of NP radiosensitizers to enhance the radiotherapeutic outcome specifically within tumor so that a greater probability of tumor control can be achieved for an acceptable risk of normal tissue complications, or radiation doses can be reduced and further spare patients from side effects.^[^ ^1^, ^7^, ^8^, ^9^, ^10^, ^11^, ^12^ ^]^ However, their dominant mechanistic function is a matter of debate.^[^ ^8^ ^]^ Initially, metal NP radiosensitization was motivated by physical mechanisms associated with IR interactions such as the photoelectric effect, Auger electron emission, pair‐production, Compton scattering, and X‐ray fluorescence, with varying cross‐section dependencies on atomic number and/or X‐ray energy.^[^ ^13^, ^14^, ^15^, ^16^ ^]^ The localized energy deposition can result in increasing direct DNA damage or local generation of ROS. However, it is now well recognized that chemical and biological mechanisms also play a critical role.^[^ ^17^ ^]^ Due to the strong energy dependence of the physical mechanisms, it is important in the current work that cells have been irradiated with a clinical 6 megavolt (MV) X‐ray spectrum, rather than a low‐energy lab‐based X‐ray source. The high energy source used here substantially reduces the probability of the photoelectric effect.

A critical aspect of metal NP radiosensitization is the mechanism by which they promote the generation of reactive species, especially ROS. There is clear evidence that the NP physicochemical properties contribute to promotion of chemical‐mediated mechanisms. Production of ROS is dependent on many variables and characteristics, such as an NP's composition, size, shape, and surface chemistry, that also governs the NP's biocompatibility, toxicity, and cellular uptake.^[^ ^18^, ^19^, ^20^, ^21^, ^22^ ^]^ The surface coating and charge also control NP stability and NP uptake, and can promote an increase in ROS production.^[^ ^23^ ^]^ The localization of radiation‐induced damages and increased levels of ROS generation within cells can overwhelm antioxidant and redox equilibrium, triggering oxidative stress, biomolecular damages, and cell death.^[^ ^24^, ^25^, ^26^, ^27^ ^]^

Biological mechanisms involved in NP radiosensitization are very complex with substantial cell line and individual cell heterogeneity.^[^ ^28^ ^]^ Cancer cells are innately heterogeneous and have a high variation in protein expression within the same population.^[^ ^29^, ^30^ ^]^ In bulk analysis, subpopulations can disproportionally affect measurements, potentially leading to incorrect conclusions.^[^ ^31^ ^]^ Analysis at a single‐cell level can promote understanding of NP‐uptake heterogeneity and the resulting impact on cells, for example, with respect to metabolism and cell size, which would not be identified by bulk analytics.^[^ ^32^ ^]^ Despite the immense interest in targeting NPs toward cancer cells, there is disproportionally little information available on factors that direct NP uptake at the individual cell level yet can drive macroscale behavior. This can only be reasonably achieved through single‐cell analysis, and has potential to divulge critical cell and NP attributes that enhance efficacy of targeted delivery.^[^ ^31^ ^]^ With respect to the current example of radiosensitization reported here, mechanistic understanding is unlikely to be fully realized unless single‐cell NP‐dose responses are resolved.

We propose that the interaction of the “radiosensitizing” NP with cells (i.e., biological mechanisms) is likely to be more important than the interaction of X rays with the NP (i.e., the physical mechanisms). For instance, if NPs alter cell regulation and impede DNA damage repair, rather than enhance the amount of DNA damage inflicted, “sensitization” would not be restricted purely to insult by X rays. In this context, we tested this proposition by also measuring sensitization of cells to a radiomimetic chemotherapeutic agent, neocarzinostatin (NCS).^[^ ^33^, ^34^, ^35^, ^36^ ^]^ NCS is a chromoprotein antibiotic that acts as a radiomimetic chemotherapeutic and comprises two components; a labile chromophore component that cleaves DNA by removal of hydrogen atoms from deoxyribose sugar and a protein component that stabilizes the chromophore.^[^ ^37^, ^38^ ^]^

We further sought to differentiate the role of NP surface chemistry on radiosensitization by comparing gold NPs with vastly different surface chemistries (i.e., polyethylene glycol [PEG] coating with or without protein conjugation, or a silica coating). However, it is extremely difficult to quantitatively compare efficacy of different NP formulations since cell uptake is extremely sensitive to NP composition and interfacial properties. Consequently, comparisons are routinely made with comparable coculture conditions yet provide ambiguous data when NPs have substantially different uptake either for different NP formulations or different cell lines. A general challenge to compare one NP to another in terms of biological impact such as toxicity or therapeutic effect persists in the sense of quantitative comparisons of not just exposure, but also uptake. NP uptake can be analyzed through bulk analysis (such as with inductively couple plasma mass spectrometry, but provides cell‐population averages and is destructive.^[^ ^39^ ^]^ For analysis at the single‐cell level, NPs are often fluorescently‐labeled with reporters for microscopy or flow cytometry, but this can affect cell uptake.^[^ ^40^ ^]^ In addition, fluorophores are susceptible to quenching, spectral overlap or uncoupling^[^ ^41^ ^]^ and are generally not quantitative.

Here, we present quantitative analysis at the single‐cell level determined with synchrotron X‐ray fluorescence imaging of the association of different NPs in two cell lines and demonstrate the value of single‐cell statistics in conducting comparisons of the true cellular impact of NPs in terms of a DNA damage repair marker (*γ*H2AX). In this study, we compared a gold NP coated with PEG (AuPEG); an actively targeted gold NP conjugated with PEG and human transferrin (AuT), which enhances cell uptake; and a gold‐core/silica‐shell NP (AuSiO_2_). The AuSiO_2_ NP conceptually promotes chemical mechanisms of radiosensitization either through the dissolution of *O*‐rich molecules to enhance ROS generation or catalyze ROS formation at their surface.^[^ ^42^ ^]^ The silica coating is also sufficiently thin to allow the majority of energetic electrons to escape from the gold core into the local environment to produce ROS. These NPs were tested in a prostate cancer cell line, PC‐3, and a head and neck cancer squamous cell carcinoma line, SCC‐1 (derived from the floor of the mouth of a male patient). In both cell lines and each insult (radiation or NCS), DNA double strand break (DSB) repair was analyzed by single‐cell analysis. The effect of the NPs was also assessed in terms of the common clonogenic assay to test for the proliferative ability of the cell population after insult. Understanding the physicochemical and radiobiological mechanisms involved in metal NP radiosensitization leads to opportunities in developing an NP that optimizes therapeutic effects.

## 2 Results and Discussion

### 2.1 Quantitative Comparisons of NP Association

**Figure 1:**
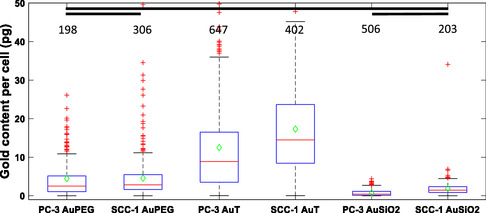
Nanoparticle association is highly variable within cell populations and between nanoparticle coatings. Single‐cell data for gold NP association per cell in picograms (pg) for AuPEG, AuT, and AuSiO_2_ NPs in PC‐3 and SCC‐1 cell lines respectively. Note however that the coculture concentrations varied between each nanoparticle. Values above each condition correspond to the number of cells analyzed for each condition. All comparisons were statistically different (one‐way analysis of variance, ANOVA; *p* < 0.05) except for the comparisons marked by black lines (*p* > 0.05).

Quantitative analysis of three NPs (AuPEG, AuT, and AuSiO_2_) across two cell lines (PC‐3 and SCC‐1) showed highly variable association across and within cell populations (**Figure** **1** and related statistics in Table S1, Supporting Information). Transferrin led to a much greater association for both cell lines as anticipated due to targeted receptor‐mediated endocytosis through the transferrin receptor, TfR1,^[^ ^43^, ^44^ ^]^ in addition to nonspecific pathways such as caveolin‐mediated endocytosis.^[^ ^45^ ^]^ We have previously shown that these NPs are almost exclusively contained within vesicles.^[^ ^46^ ^]^ A significantly greater NP association was observed for the AuT NPs in the SCC‐1 cells compared to PC‐3, despite the average size of the PC‐3 cells being larger than the SCC‐1 cells (409 μm^2^ compared to 199 μm^2^, respectively).^[^ ^47^ ^]^ We sought to understand if this difference was due to differences in expression of TfR1 between the two cell lines. While larger cells can have greater NP uptake,^[^ ^48^ ^]^ it also depends on receptor density^[^ ^32^ ^]^ and so we undertook imaging flow cytometry to analyze TfR1 expression. While the PC‐3 cells are larger, on average, for cells of comparable size the SCC‐1 cells have a greater expression than PC‐3, except for very large cells (Figure S1, Supporting Information). Overall, the mean TfR1 fluorescence detected for PC‐3 cells (28307) was greater than SCC‐1 (25487); however, the median value for SCC‐1 (18572) was greater than PC‐3 (13605) (*p* < 0.0001, Mann–Whitney *U* test). This is due to the PC‐3 cells having some cells with much greater expression than the SCC‐1 cells for comparable size, which skews the mean value. Such observations reinforce the importance of single‐cell analysis, which can provide contradictory results compared to bulk, averaged data.

For the coculture conditions used, no statistically significant difference was measured between cell lines for either the AuPEG or AuSiO_2_ NPs. While the size of NPs can affect NP internalization,^[^ ^49^, ^50^ ^]^ size is not expected to be a dominating influence here as the differences in NP sizes (hydrodynamic diameters of 38.8, 46.5, and 21.1 nm for AuPEG, AuT, and AuSiO_2_, respectively) are modest. Uptake is expected to be primarily mediated through interfacial properties.^[^ ^51^, ^52^ ^]^

### 2.2 NP Effects on DNA Damage Repair Foci After X‐Ray Irradiation

**Figure 2:**
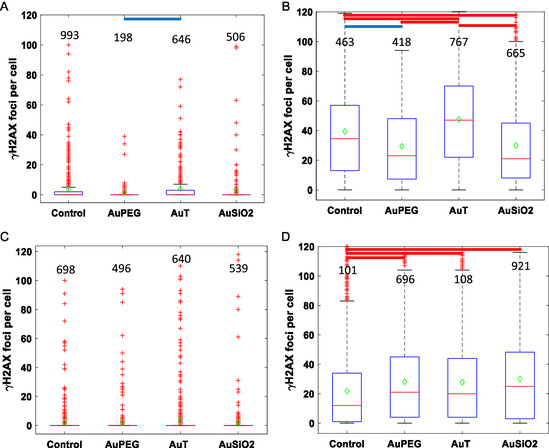
Box and whisker plots of *γ*H2AX foci per cell for A,B) PC‐3and C,D) SCC‐1 cell lines for a 0 and 4 Gy X‐ray irradiation, respectively, after incubation with AuPEG, AuT, and AuSiO_2_ NPs. Values above each condition correspond to the number of cells analyzed. A one‐way ANOVA significance test has been carried out on each dataset to determine statistical significance between the means. Red lines indicate a highly significant difference between the means (*p* < 0.0001) and blue lines indicate a statistical difference between the means (*p* < 0.05).

While multiple DNA damage response markers exist, *γ*H2AX was used as it is the most prevalent marker used for measuring DNA damage repair, formed by the phosphorylation of the histone H2AX in response to DNA DSB damage.^[^ ^53^, ^54^ ^]^ One hour postirradiation with 4 Gy from a clinical 6 MV LINAC, individual cells were stained for *γ*H2AX and imaged with confocal microscopy. Foci after NP incubation and sham or 4 Gy X‐ray irradiation are given in **Figure** **2** with corresponding statistics in Table S2, Supporting Information. Most cells exhibited no foci from the sham irradiation although some did exhibit foci, which occur spontaneously; such as from nuclease activity or from the stress induced by the sham process itself. Cells expressing *γ*H2AX foci lying beyond the box and whiskers plot were classified as outliers as described in Statistical Analysis section. For sham irradiation, only in the AuT NP sample was there a slight but statistically significant increase in the mean number of *γ*H2AX foci for the PC‐3 cells.

A substantially different radiobiological response was observed between the cell lines for the control condition not containing NPs. However, it is often not a trivial exercise to interpret *γ*H2AX foci^[^ ^55^ ^]^ as radiation damage is a stochastic physicochemical process, repair is a highly regulated biological process, and NPs can disrupt associated pathways.^[^ ^56^, ^57^ ^]^ A reduction in the number of *γ*H2AX foci can represent either an impeded DNA DSB repair mechanism or an enhanced repair.^[^ ^53^ ^]^ Head and neck SCC are characteristically radiation resistant due to enhanced DNA damage repair.^[^ ^58^ ^]^ In this regard, it is unsurprising that the SCC‐1 cells have undertaken more efficient repair at this 1 h time point and exhibit fewer foci.

NPs may be involved in increasing the local energy deposition (dose) and DNA DSBs or by downregulating repair as seen in the PC‐3 cells, where AuT NPs caused a statistically significant increase in *γ*H2AX foci compared to control (Figure 2B). Whereas, AuPEG and AuSiO_2_ NPs caused a statistically significant reduction in *γ*H2AX foci when compared to control. In SCC‐1 cells, all three NPs caused greater numbers of *γ*H2AX foci compared to control cells (Figure 2D). While the SCC‐1 cells had an approximate tenfold increase in the number of associated AuT NPs compared to the AuSiO_2_ NPs, both NPs resulted in comparable numbers of DNA damage repair foci.

The contrast of the whole‐cell population overview of the radiobiological response (Figure 2) and the drastic difference in NP uptake due to NP surface chemistry and functionalization (Figure 1) highlights the challenge in comparing one NP to another.

**Figure 3:**
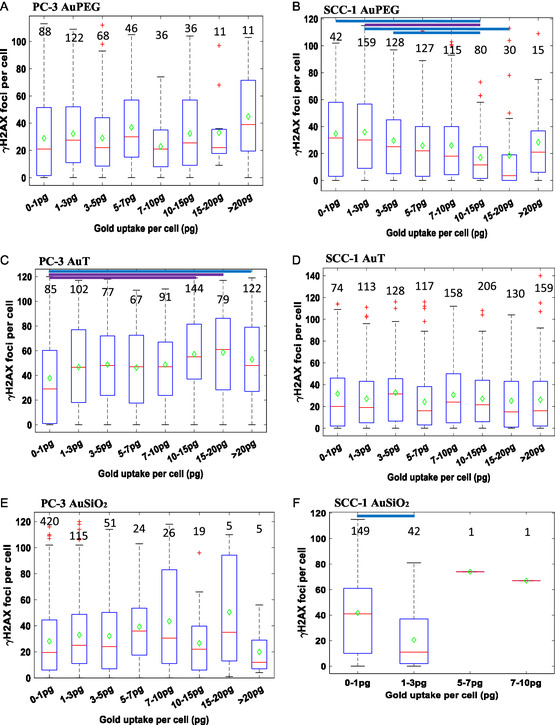
Box and whisker plot of the *γ*H2AX foci per cell of the PC‐3 and SCC‐1 cell lines after 4 Gy X‐ray irradiation, sorted by the amount of gold associated with individual cells after incubation with A,B) AuPEG, C,D) AuT, and E,F) AuSiO_2_ NPs for each cell line, respectively. Values above each condition correspond to the number of cells analyzed. A one‐way ANOVA significance test has been carried out on each dataset to determine statistical significance between the means. Purple lines indicate a statistical significance between the means (*p* < 0.001) and blue lines indicate a statistical difference between the means (*p* < 0.05).

The radiobiological response in cell subpopulations with the same number of NPs associated was investigated by comparing *γ*H2AX foci to NP association according to a number of “bins” spanning the range of NP association (**Figure** **3**, Table S3 and S4, Supporting Information). While AuPEG NPs led to a lower number of foci compared to control in PC‐3 cells (Figure 2B), no NP‐dependent trend was observed within the NP‐treated cell population (Figure 3A). For PC‐3 cells with less than 1 pg gold AuT NPs (Figure 3B), foci were fewer than the control, but were significantly greater with the association of greater numbers of NPs (e.g., *p* = 0.0009 comparing the 0–1 pg and 10–15 pg bins). For AuSiO_2_ in PC‐3 cells (Figure 3E), there appeared to be a general NP‐dose‐dependent increase in foci; however, no two comparisons were statistically significant.

In the SCC‐1 cell line, all NP conditions yielded a greater mean *γ*H2AX focus per cell (28.1, 27.9, and 30.2 foci for the AuPEG, AuT, and AuSiO_2_ NP conditions, respectively) over that of control (21.7 foci) (Figure 2D). The AuPEG NP condition interestingly had a trend of a decreasing number of *γ*H2AX foci as the NP association increased above ≈3 pg per cell, becoming statistically significant above 10 pg per cell (Figure 3B). This could indicate a dose response to the NPs that leads to either accelerated or delayed repair with an exception in cells with greater than 20 pg of NPs. This may be due to a few cells (*n* = 15% or 2.2%) representing a small population that is phenotypically disparate to the rest of the population. The amount of AuSiO_2_ NPs associated with the SCC‐1 cells was substantially lower than the other NP conditions, even though they resulted in the highest number of *γ*H2AX foci.

Overall, in the PC‐3 cell line for AuT NPs, there was a slight trend of increasing *γ*H2AX foci with gold association. Whereas, in the SCC‐1 cell line, an opposite trend occurred for AuPEG and AuSiO_2_ NPs, or no significance was observed in the AuT condition. This highlights that different cell lines interact with certain NPs and radiation very differently in radiosensitization and the tailoring of specific NPs to sites may benefit therapeutic results. This has been demonstrated in other studies where platinum‐based NPs had no radiation enhancement in the T47D and MDA‐MB‐231 breast cancer cell lines^[^ ^59^ ^]^ compared to the HeLa cervical cancer cell line^[^ ^60^ ^]^ despite the same conditions and NP formulation.

The power of the analytical methodology used here to compare equivalent NP association with cells rather than equivalent coculture conditions deserves emphasis. For instance, we are able to count how *γ*H2AX foci are expressed by cells with, for example, 1–3 pg of AuT NPs after 4 Gy irradiation. In this example, PC‐3 cells express an average of ≈47 foci compared to ≈27 foci for SCC1 cells. Similarly, we observe that the average number of foci is ≈36 for SCC1 cells with 1–3pg of AuPEG NPs yet only ≈21 foci for 1–3 pg of AuSiO_2_ NPs. The gold content is equivalent for each of these conditions, yet average *γ*H2AX expression is substantially different from which we can conclude is due to cell‐line‐dependent traits and NP surface chemistry. However, a limitation in this study is that unequal concentrations of the different NP types were used in the coculture with cells. The biological response of a cell subpopulation internalizing a specific number of NPs may differ to a subpopulation of cells internalizing the same number of NPs, but under a different coculture concentration. This could conceivably give rise to an additional dimension of investigation of nano–bio interactions.

### 2.3 Effect of NPs on Cell Clonogenicity After Irradiation

**Figure 4:**
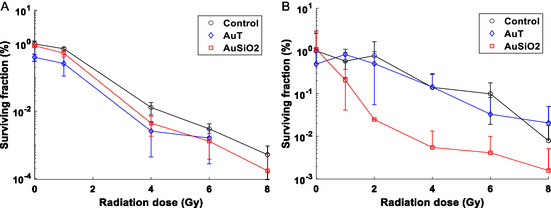
Clonogenic assay of the A) PC‐3 and B) SCC‐1 cell lines demonstrating the survival curves after NP incubation and irradiation. *n* = 5.

The clonogenic assay is the gold standard in assessment of the overall survival of individual cells by their proliferative potential to divide and form substantially‐sized colonies^[^ ^61^, ^62^ ^]^ after insult with radiation.^[^ ^63^ ^]^ *γ*H2AX foci analysis provides an indication of radiation damage, activation of repair mechanisms, and is extremely valuable in single‐cell analysis but the clonogenic assay provides the overall longer‐term survival profile of cells populations. Clonogenic curves were collected for both the PC‐3 and the SCC‐1 cell lines after AuT or AuSiO_2_ NP incubation (**Figure** **4**), representing the NPs taken up the most and least, respectively. For each cell line, all conditions were normalized by the 0 Gy control condition with no NPs, classified as the plating efficiency, for the determination of the surviving fraction. AuT NPs show a reduction in the proliferative capacity of both cell lines (i.e., data for 0 Gy), though only significant in the PC‐3 cell line; while no effect is observed for the AuSiO_2_ NPs.

PC‐3 cells are substantially more sensitive to radiation compared to SCC‐1, demonstrated by the control curves in the clonogenic data. For PC‐3 cells, both NPs provided a modest reduction in the surviving fraction with radiation, which was significant only at 4 Gy (*p* = 0.03, *p* = 0.04 for AuT and AuSiO_2_, respectively). This contrasts data for the number of *γ*H2AX foci counted in the AuT and AuSiO_2_ NP conditions (Figure 2B), where AuT NPs led to a greater number of repair foci, and AuSiO_2_ NPs led to a lower number. This suggests that NPs may contribute to reducing proliferative capacity of cells by mechanisms other than by DNA damage or through impairing cell repair mechanisms. This data highlights that DNA repair foci at a specific time point are not a good sole marker for predicting cell survival or the most efficacious radiosensitizer. The physical presence of NPs has been shown to affect subcellular processes and signaling,^[^ ^56^, ^57^, ^64^ ^]^ which may explain this observation.

In SCC‐1 cells with irradiation, AuT NPs did not impact cell survival (Figure 4B). The AuSiO_2_ NPs however led to much greater reduction in survival compared to control despite having far fewer AuSiO_2_ NPs associated compared to AuT (Figure 1). The *γ*H2AX assay exhibited comparable numbers of foci for each NP condition (Figure 2D) and again shows that radiosensitization and longer‐term radiobiological response cannot be predicted with DNA damage repair foci measured by *γ*H2AX. We interpret the clonogenic data presented in Figure 4 as that the AuSiO_2_ NPs exert a cell‐specific synergistic effect in combination with radiation, not due to interaction of the X rays with the Au NP, but due to the biological impact of the NP on the cell due to the silica coating. If a dose enhancement was to occur due to the physical processes involved in X‐ray interactions with gold, we could expect greater impact for the AuT NPs due to their greater association with cells (Figure 1), and also that the physical aspects of dose enhancement are not cell specific. Consequently, the quantity of gold associated with cells in each condition is not the determining factor in overall cell survival. Rather, the NP coating and its interfacial interactions dominate. This could be either through altering local chemical environment, interfacial catalytic formation of ROS, or changing cell regulation. These possibilities are further investigated later.

### 2.4 ROS Generation from NPs and Radiation

**Figure 5:**
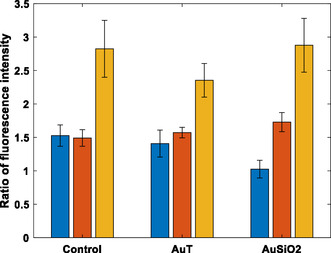
ROS is greater in cells after irradiation but nanoparticles have a minor effect on ROS generation. The ratio of the average fluorescence intensity after receiving 4 Gy compared to 0 Gy data; indicative of the ratio of the reactive oxygen species generation with AuT and AuSiO_2_ NP conditions in solution only (blue), and in PC‐3 (orange) and SCC‐1 cells (yellow). “Control” is with radiation and no nanoparticles. *n* = 2.

To test the role of ROS generation in promoting cell stress, the DCFDA assay was applied. AuSiO_2_ NPs led to a massive increase in ROS in cell media (with no cells) compared to the other NPs even without radiation (Figure S2B, Supporting Information). However, this was not apparent in either cell line (Figure SC2 and D, Supporting Information), presumably due to ROS being mediated by cellular processes and many fewer NPs being associated with the cells compared to being in solution. AuSiO_2_ NPs have the capacity to promote the formation of ROS but their numbers appear to be too few to show a notable effect in cells and any relative increase in ROS due to the radiation and NPs is only modest (**Figure** **5**). Figure 5 demonstrates the ratio of the average 4 Gy fluorescence intensity to the average of the 0 Gy fluorescence intensity for the conditions in solution and in both cell lines, taken from the data in Figure S2, Supporting Information. In all conditions, the ratio is above 1, indicating that the irradiated samples have a higher ROS generation than the nonirradiated samples. The SCC‐1 cells demonstrate a larger increase in the ratio of fluorescence intensity than other conditions and thus, the impact of ROS generation may be more influential on the impact of sensitization after radiation than the PC‐3 cell line. This could be due to the inherent resistance to ROS of each cell line, where this is regulated by the level of glutathione and catalase activity.^[^ ^65^, ^66^ ^]^ The ratio of ROS generation was higher with AuSiO_2_ NPs in both cell lines. Though not definitive, the AuSiO_2_ NPs appear to promote the generation of ROS and the increase in sensitization seen in the clonogenic assays is likely partly due to this enhancement of ROS generation. In other work, dissolution of silica from the AuSiO_2_ NPs was proposed to be responsible for ROS enhancement as nanoscale silica has been shown to dissolve in solution,^[^ ^67^ ^]^ which accelerates in biological media.^[^ ^68^ ^]^ This dissolution of silica would likely promote formation of ROS and may contribute to the radiobiological affects shown in this article.

In both cell lines, AuSiO_2_ NPs demonstrated a reduction in clonogenic survival despite having much lower association with cells when compared to the AuT NP. Inhibition of repair processes or a disruption in cell regulation may be responsible for this overall damage enhancement. Indirect damage, primarily caused by interactions of IR with NPs, may also promote chemical‐based mechanisms, primarily via the enhancement of ROS generation.^[^ ^46^, ^69^, ^70^ ^]^ The combination of the localization of radiation‐induced damages and increased levels of ROS within cells can trigger oxidative stress, biomolecular damage, and cell death.^[^ ^71^ ^]^

### 2.5 Radiomimetic Drug Exposure

The gold silica NPs reduced clonogenicity with radiation (Figure 4B), which did not appear to be due to the physical interaction of the X rays with gold, or otherwise it would be expected that the AuT NPs would have a greater impact due to a greater association with cells. Consequently, the ability of the different NP coatings to generate ROS was tested, showing a large effect in solution, but only minor differences within cells when irradiated. In addition to these physical and chemical mechanisms, cells may also be sensitized to insult through biological mechanisms since NPs can affect cellular homeostatic function^[^ ^72^, ^73^ ^]^ and impact DNA damage response. To discriminate this, NP‐treated cells were also treated with the radiomimetic chemical agent, NCS.^[^ ^74^, ^75^ ^]^ NCS induces cell DNA damage from the extraction of hydrogen atoms from deoxyribose sugar and by inhibition of DNA synthesis.^[^ ^76^ ^]^ Furthermore, ROS are promoted and assist in the cleavage of cell DNA during NCS incubation^[^ ^34^, ^75^ ^]^ analogous to the mechanistic action of IR. If the mechanisms of NPs enhancing the effects of radiation are simply due to biological interactions of the NP with cells (rather than the interaction of X rays with NPs), sensitization should occur for chemical agents such as NCS as well as for X‐rays. And as the cause of cell death from NCS use is analogous to the direct and indirect action on cells during radiation therapy, investigation and comparison of the impact and possible mechanisms of metal NPs can be further investigated.

**Figure 6:**
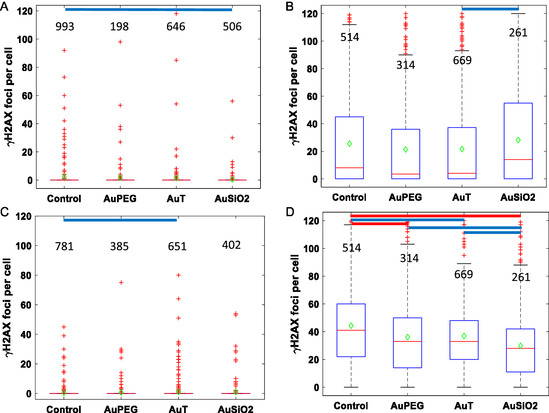
Box and whisker plot of the *γ*H2AX foci per cell of the A,B) PC‐3 and C,D) SCC‐1 cell lines for A,C) cells not exposed to the radiomimetic drug, NCS, and B,D) exposed to 10 μg mL^−1^ NCS, respectively, after incubation with AuPEG, AuT, and AuSiO_2_ NPs. Values above each condition correspond to the number of cells analyzed. A one‐way ANOVA significance test has been carried out on each dataset to determine statistical significance between the means. Red lines indicate *p* < 0.0001 and blue lines *p* < 0.05.

Cellular damage and subsequent repair recruitment mechanisms in PC‐3 and SCC‐1 cells by NCS insult were analyzed by fluorescent marking of the *γ*H2AX protein and data are presented in box‐and‐whiskers plots (**Figure** **6**) with associated statistical significance between the means. The associated statistics of the *γ*H2AX foci including the mean, median, and standard deviation is listed in Table S5, Supporting Information. With respect to the *γ*H2AX assay, no NP led to a statistical difference in foci when cells were exposed to NCS in PC‐3 cells. However, for SCC‐1 cells, every NP led to a significantly lower number of foci compared to the cells exposed to NCS only. This was the opposite of what was observed in SCC‐1 cells after irradiation (Figure 2D). It is important to differentiate that for the radiomimetic drug, its action is mediated by biological mechanisms for uptake and transport into the nucleus whereas for radiation, the ionizing events caused by X rays is independent of biology. In this context, fewer foci for the NCS treatment could mean NPs inhibited the action of NCS or promoted faster DNA damage repair. The PC‐3 cell line presented more foci than SCC‐1 after irradiation, whereas in the radiomimetic study, the SCC‐1 cell line exhibited more foci. This also reinforces the notion that different cell lines respond heterogeneously from radiation and drug treatment to one another.

A notable difference between the NCS study and radiation is observed from the skew to the lower end of the data distribution, visualized from the lower quartile of the box and the median values. This would be likely due to a nonhomogeneous distribution and uptake of the drug in cells when compared to radiation. Influence of NPs on the *γ*H2AX foci from exposure of NCS was shown to be very minimal in the PC‐3 cell line.

It is expected that during and after NP internalization, surface interactions occur between NPs and biomolecules within vesicles, often causing aggregation.^[^ ^77^, ^78^, ^79^ ^]^ Interactions between NCS and NPs may be possible, passivating the functionality of NCS. While this could act as an explanation of the observations made for the SCC‐1 cell line, minimal differences between the control and NP conditions were observed for the PC‐3 cell line after NCS exposure and it is therefore difficult to make this conclusion. It may be that translocation of NCS into the nucleus or resulting ROS responsible for DNA damage is greater in the SCC‐1 cell line naturally and NPs limit this interaction either through scavenging ROS or impeding translocation. The SCC‐1 cells appear much more adept in regulating ROS than PC‐3 as the DCFDA measurements indicated many more ROS were produced in SCC‐1 cells after 4 Gy irradiation than in PC‐3. However, this did not translate to any enhancement in cell‐killing measured by clonogenic assay for the AuT NPs. The regulation of ROS intracellularly by the NPs also does not appear to be a reasonable explanation for these observations. The most reasonable explanation for the observations appears to be that AuSiO_2_ NPs impede NCS translocation into the nucleus, especially for the SCC‐1 cells. This interpretation would then explain why fewer foci are observed in cells, after exposure to AuSiO_2_ NPs and treatment with NCS.

**Figure 7:**
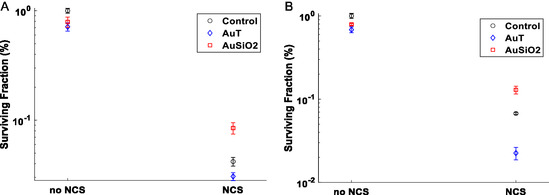
Clonogenic assay of A) the PC‐3 and B) SCC‐1 cell lines demonstrating survival after incubation with NPs and the radiomimetic drug, neocarzinostatin, at 10 μg mL^−1^. *n* = 3.

Clonogenic assays were conducted for both the PC‐3 and the SCC‐1 cell lines after AuT or AuSiO_2_ NP incubation and subsequent treatment with NCS at a concentration of 10 μg mL^−1^ (**Figure** **7**). For each cell line, all conditions were normalized by the non‐treated condition containing no NPs for the determination of the surviving fraction. For cells with no exposure to NCS, the NPs had a reduced surviving fraction compared to control (within the PC‐3 cell line *p* = 0.01 and *p* = 0.04 and the SCC‐1 cell line *p* = 0.002 and *p* = 0.02 for AuT and AuSiO_2_ NPs, respectively). This reduction in cell survival was more significant for the AuT NP condition, which was also reflected in the clonogenic assay from the radiation study. It appears that AuT NPs have an impact on overall cell survival after incubation, but the exact mechanisms are not known and were not considered in this study.

For cells exposed to NCS, cell survival reduced in the AuT NP condition but increased in the AuSiO_2_ NP condition in both cell lines when compared to the control (no NPs). In both cell lines, NP conditions exposed to NCS were statistically different to the control condition except between the control and AuT condition in the PC‐3 cell line (*p* = 0.26). This would indicate that AuT NPs are typically contributing to a reduced cell survival by promotion of NCS‐related mechanisms and the AuSiO_2_ NPs are inhibiting this activity, leading to increased cell survival. This observation is consistent with the interpretation that AuSiO_2_ NPs are having significant impact on translocation of NCS into the nucleus of SCC‐1 cells. A summary of the key observations for all conditions is provided in **Table** **1**.

**Table 1:** A summary of the results for each cell line, for each nanoparticle tested and assays performed.

	Nanoparticle uptake			*γ*H2AX after IR cf. IR alonea)			*γ*H2AX after NCS cf. NCS alone		
	PEG	AuT	SiO_2_	PEG	AuT	SiO_2_	PEG	AuT	SiO_2_
PC3	**••**	**••••**	**•**	**↓**	**↑**	**↓**	**–**	**–**	**–**
SCC1	**••**	**•••••**	**•**	**↑**	**↑**	**↑**	**↓**	**↓**	**↓**
				Clonogenic response. NP's caused treatment: sensitivity (**↑**); or resistance (**↓**)					
PC3					**↑**	**↑**		**↑**	**↓**
SCC1					**–**	**↑**		**↑**	**↓**

a) IR: ionizing radiation; NCS: neocarzinostatin.

When directly comparing the surviving fraction of cells exposed to 10 μg mL^−1^ of NCS to the clonogenic assay after radiation exposure, NCS is approximately equivalent to 3 Gy in the PC‐3 cell line and 6 Gy in the SCC‐1 cell line. From the *γ*H2AX foci after NCS exposure, there was a greater count in the SCC‐1 cell line over the PC‐3 cell line also. This further highlights the notion that each cell line responds quite distinctly to external insults. The PC‐3 cell line is more susceptible to damage from X‐ray radiation and the SCC‐1 cell line is more susceptible to damage from the radiomimetic drug. When considering the addition of gold NPs, the AuT and AuSiO_2_ NPs have demonstrated their impact with radiation and NCS. AuT NPs appear to promote DNA damage from radiation and may lead to radiosensitization in the PC‐3 cell line. While in NCS studies, AuT NPs promote cell death despite little variance in DNA damage. AuSiO_2_ NPs promote sensitization of radiation in both cell lines, but are more pronounced in the SCC‐1 cell line. However, in the NCS study, the AuSiO_2_ NPs result in fewer DSB repair foci, leading to the inhibition of NCS function and improved cell survival. The physical and chemical interactions of AuSiO_2_ NPs in cells likely impede NCS action, promoting cell survival. NPs impact the regulatory behavior of the cellular environment, affecting the cell response and fate from DNA damage.

## 3 Conclusions

AuSiO_2_ NPs resulted in an increase in the expression of *γ*H2AX foci after irradiation and substantial reduction in colony formation in SCC1 cells. Despite the AuT NP association with cells being approximately tenfold greater, an increase in *γ*H2AX was not as large and there was no change in colony formation compared to radiation alone. These data indicate that any radiosensitization effect is not dependent upon the amount of the high‐Z element (gold) associated with each cell. The radiosensitization action of the AuSiO_2_ NPs did not appear to be an additive stress response from cells since the AuSiO_2_ NPs induced a protective effect when the cells were exposed to the radiomimetic drug. Imparting a protective effect from the radiomimetic drug for the AuSiO_2_, NP‐treated SCC1 cells corresponded with expression of fewer *γ*H2AX foci. AuT treatment led to an even greater reduction in foci, but conversely corresponded with a sensitization effect to the radiomimetic drug. Whole cell‐population *γ*H2AX expression is not a good predictor of cells’ survival and colony‐forming ability. According to the clonogenic assays, the greatest radiosensitization effect was induced in the SCC‐1 cells by the NPs taken up the least (i.e., AuSiO_2_ NPs and not the AuT NPs). This indicates that the radiosensitization is not necessarily related to the extent of NP uptake, but rather the extent of biological impact which is directed by the surface interactions.

Contrary to the proposition that high‐Z NP composition is required for metal NP radiosensitization, the gold content appeared irrelevant to the sensitization/protective action of the NPs and provides evidence that under these experimental conditions, the physical processes involved in radiosensitization are negligible. Rather, biological response to radiation or radiomimetic insult is dominated by NP surface chemistry and the specific cell lines’ traits in terms of susceptibility to the treatment, ROS regulation, and/or NP impact on cellular processes such as molecular transport and translocation and protein expression, i.e., the radiosensitization effect is dominated by NP–cell interactions rather than X‐ray NP interactions. Further studies will identify which pathways are manipulated by NPs and link specific surface chemistry traits to these responses with the aim to optimize opportunities in potentiation of cancer therapies.

## 4 Experimental Section

4.1

4.1.1

##### NP Preparation

Gold seed solution was synthesized using the Turkevich method^[^ ^80^ ^]^ with gold (III) chloride trihydrate (Sigma‐Aldrich) and sodium citrate solution (Sigma‐Aldrich) for use as a base for the three NPs. AuPEG NPs were synthesized by adding 400 μL mixture of short PEG (458.6 g mol^−1^, Polypure) and long PEG (5000 g mol^−1^, Rapp Polymere) at a molar ratio of 2:1 for 12 h at 4 °C to 400 μL gold seed. Between each NP synthesis step, the NP solution was centrifuged at 14 000 g to remove excess reagents and discontinue the reaction. Gold–transferrin (AuT) NPs were synthesized by cross‐linking 750 μL of the AuPEG NPs with a 150 μL mixture of 0.4 M 1‐ethyl‐3‐(3‐dimethylaminopropyl) carbodiimide (Sigma‐Aldrich) and 0.1 M N‐hydroxysuccinimide (Sigma‐Aldrich) for 5 min at room temperature. An amount of 20 μL of 500 μg mL^−1^ holo‐human transferrin (Sigma‐Aldrich) was then added. AuSiO_2_ gold‐core/silica‐shell NPs were developed using a modified version of the method developed by Liz Marzan et al.^[^ ^81^ ^]^ by adding 25 μL of 1 mM 3‐aminopropyltrimethoxysilane (Sigma‐Aldrich) to 5 mL of gold seed under vigorous magnetic stirring and left to stand for 15 min. An amount of 200 μL of 0.54% sodium silicate solution (Sigma‐Aldrich) was added under vigorous stirring and left under light stirring for 24 h.

The NP hydrodynamic sizes were characterized by dynamic light scattering (Malvern Instruments Zetasizer Nano ZS) and absorbance peaks were measured with UV–vis spectrometry (Thermo Evolution 201 UV–Vis Spectrophotometer) to confirm successful synthesis and determine concentration as per the method of Haiss et al.^[^ ^82^ ^]^ NPs with a hydrodynamic diameter of 18 ± 3.5, 38.8 ± 15, 46.5 ± 17, and 21.1 ± 7 nm were measured for the gold seed, AuPEG, AuT, and AuSiO_2_ NPs, respectively.

A JEOL JEM‐2100 F‐HR transmission electron microscope at 200 kV accelerating voltage was used to visualize the size and shape of all NPs and the silica shell on the surface of the gold core for the AuSiO_2_ NP (Figure S3, Supporting Information). Copper grids were prepared for analysis by dilution of the NP samples with Milli‐Q water until the solution was clear. One to two drops of the diluted solution was added to the grid and left to dry.

##### Cell Culture

The PC‐3 (ECACC, 90112714) and an SCC‐1 (Merck Millipore) were used. Both cell lines were cultured in Roswell Park Memorial Institute (RPMI) media with 0.3 g L^−1^ L‐glutamine (Life Technologies), supplemented with 2% penicillin–streptomycin (Life Technologies) and 10% fetal bovine serum (Life Technologies) and kept in a humidified incubator at 37 °C and 5% CO_2_.

For confocal analysis, ≈150 000 cells per well were plated in a flexiPERM 8‐well slide (Sarstedt) and for clonogenic analysis, ≈4 × 10^5^ cells were plated in each well of a 6‐well plate. After overnight cell adhesion, an initial gold concentration of 0.46, 0.26, and 0.093 μmol mL^−1^ for AuPEG, AuT, and AuSiO_2_ NPs were incubated with cells at 37 °C for 2 h and washed with phosphate buffered saline (Thermo Fisher Scientific) (PBS) and fresh media was added prior to exposure to radiation or NCS. These concentrations were applied to other experiments. As the tolerance of cells to NPs with different coatings is variable, the NP coculture conditions used here were at a ratio of ≈4:2:1, which was based on qualitative observation of the degree of biocompatibility of each NP (i.e., the PEG NPs were most biocompatible and thus used at the highest concentration).

##### X‐Ray Irradiation

Samples were transported to the Radiation Oncology Department of the Lyell McEwin Hospital and irradiated under reference conditions using a 6 MV photon beam from a Varian Clinac iX Linear Accelerator (Varian Medical System, Palo Alto, CA). The linear accelerator was calibrated using the International Atomic Energy Agency TRS‐398 protocol, and the radiation dose output was checked on the day of irradiation with a Daily QA 3 device (Sun Nuclear, USA). Well plates were placed on 1.3 cm thick solid water buildup sheets so that the cells were positioned at the depth of maximum dose and 5 cm of solid water buildup sheets were placed on top to provide full scattering conditions. Empty wells were filled with media to generate side scatter. The well plates were irradiated with the gantry positioned at 180°, source to surface distance of 100 cm and a 20 × 20 cm^2^ field size. Irradiation doses of 0, 1, 2, 4, 6, and/or 8 Gy at a dose rate of 300 cGy min^−1^ were used.

##### Radiomimetic Drug Exposure

Samples were treated with the radiomimetic chemotherapeutic drug, NCS from *Streptomyces carzinostaticus* (Sigma‐Aldrich) (NCS). An amount of 100 μL of 10 μg mL^−1^ NCS was added to the cells and incubated for 30 min at 37 °C.

##### Immunofluorescence Staining

After treatment, cells were washed in PBS and fixed with 95% ethanol (Sigma‐Aldrich) and 5% acetic acid (Sigma‐Aldrich) on ice for 10 min. Cells were washed again with PBS and permeabilized in PBS containing 0.5% Triton X‐100 (Fisher Scientific) for 15 min at 37 °C. After permeabilization, cells were subsequently washed and incubated at 37 °C in PBS containing 5% goat serum for 1 h to block nonspecific binding. Anti‐*γ*H2AX antibody (Abcam) was used at a 1/500 dilution in PBS containing 1% goat serum and incubated for 1 h at 37 °C. After washing cells with PBS, cells were incubated with goat anti‐mouse IgG Alexa Fluor 488 sary antibody (Abcam) at a 1/500 dilution in 1% goat serum for 1 h at 37 °C. 4′,6‐diamidino‐2‐phenylindole (Thermo Fisher Scientific) (DAPI) was added to the fixed cells at a 1/10 000 dilution for 10 min at room temperature to allow identification of cell nuclei during imaging. Cells were rinsed thoroughly with Milli‐Q water prior to confocal microscopy being performed.

##### Confocal Microscopy

Acquisition of the fluorescent image sets was performed with a Zeiss LSM 710 laser scanning confocal microscope (Carl Zeiss). Images were acquired with a 20× objective with a 405 nm laser for the DAPI channel, 488 nm laser for the *γ*H2AX channel, and 560 nm laser for the thymidylate synthase (TS) channel. Image dimensions were 7168 × 1024 pixels, corresponding to image size of 2.9 × 0.42 mm giving x and y resolutions of 0.415 μm. Z stacks were utilized for each condition using 2 μm slice width.

##### Imaging Flow Cytometry

The PC‐3 and SCC‐1 cell lines were stained for Cluster of Differentiation 71 (CD71), also known as transferrin receptor 1 (TfR1) with fluorescein isothiocyanate mouse antihuman CD71 (BD Biosciences) as per the instructions provided in the product sheet at 1/500 dilution for 30 min and Hoechst (Thermo Fisher Scientific) at 1/10 000 dilution for 10 min. The cells were then analyzed using an ImageStreamX Mark II multispectral imaging flow cytometer (Amnis) with a 488 nm laser for TfR1 and 405 nm laser for Hoechst. Approximately 10 000 cells were measured and individual cells were isolated from doublets, triplets, or debris by gating within the Image Data Exploration and Analysis Software (IDEAS). A negative control was implemented for cells that remained unstained for TfR1 and used for background subtraction of the data. Single‐color compensation was performed within the IDEAS software so that accurate fluorescence intensity was assured. Hoechst was used to evaluate cell size within the IDEAS software.

##### Quantitative Analysis of NP Uptake by X‐Ray Fluorescence

After acquisition of confocal image sets, the wells were washed with Milli‐Q water before addition of CuSO_4_ (Sigma‐Aldrich) at a dilution of 1:10 000 for 1 h. After washing with Milli‐Q water, wells were dried in preparation for X‐ray fluorescence (XRF) analysis. XRF inorganic elemental analysis was conducted at the Australian synchrotron X‐ray fluorescence microscopy beamline.^[^ ^83^ ^]^ A step size of 2 × 2 μm was used, with a corresponding pixel dwell time of 4 μs to acquire high‐resolution images. Data quantification was performed using reference spectra of known composition and concentration from thin foil materials to yield gold elemental maps from the raw XRF data using GeoPIXE.^[^ ^84^ ^]^ The elemental map images were saved in a tiff format, displaying pixel intensity values in ng cm^−2^.

##### Image and Data Analysis

**Figure 8:**
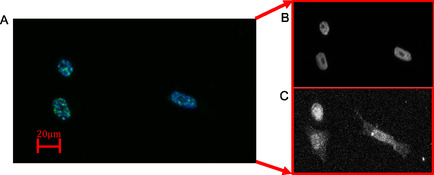
Alignment and overlay methodology used for image data analysis with an example of manual cell outline and determination. A) Confocal maximum projection image for both the DAPI (blue) and *γ*H2AX (green) channels. The corresponding B) DAPI image identifies the nuclear region and C) TS image identifies the whole cell boundary.

The 8‐bit grayscale raw confocal images were exported from Zen Black software (Carl Zeiss). The copper elemental map from XRF was used to overlay cells to the subsequent TS confocal images and by extension, onto the XRF gold elemental map to identify subsequent NP association with cells. TS is a ubiquitous stain and was used as an accurate representation of the whole cell region. These overlays were performed in Adobe Photoshop CC (2020 Adobe Systems Incorporated). The aligned image sets were exported as tag image file format files to MATLAB (MathWorks R2018a) for cell size and gold association analysis. *γ*H2AX was measured within the nuclear boundary of the DAPI image and gold amount was measured using the whole cell boundary from the TS image. Cell masks were manually defined in MATLAB (**Figure** **8**) using a combination of custom code^[^ ^31^ ^]^ and in‐built image processing features. The pixel values were converted into their physical size in cm^2^ to allow the integration process to yield a value in ng. Thresholds were included within the analysis process to identify and exclude apoptotic cells.

##### Clonogenic Analysis

After treatment, cells were put into suspension using trypsin–ethylenediaminetetraacetic acid (Life Technologies) and the number of cells was counted a minimum of three times. The cells were resuspended at variable cell numbers depending on radiation dose and cell line in a petri dish in up to five replicates (**Table** **2**). The cells were maintained until the colonies in the control sample were sufficiently large enough (≈25–50 cells after 14 days). Each well was washed with PBS before 0.5% weight/volume crystal violet (Sigma‐Aldrich), diluted with 100% methanol (Sigma‐Aldrich), was added for 5–10 min to fix and stain the colonies. The solution was removed, and each petri dish was rinsed in water and left to dry. The colonies were counted using a Leica Wild M8 stereomicroscope, using a photo of each sample as a reference guide.

**Table 2:** Clonogenic assay plating numbers after radiation exposure.

Cell line	0 Gy	1 Gy	2 Gy	4 Gy	6 Gy	8 Gy
PC‐3	100	200	300	500	800	1000
SCC‐1	200	300	500	750	1000	1300

For analysis of clonogenicity using NCS after AuT and AuSiO_2_ NP incubation, the plating number used for the PC‐3 cell line was 400 cells where no NCS was added and 800 cells in the NCS samples. For the SCC‐1 cell line, 1500 cells and 3000 cells were used for no NCS added and NCS, respectively.

##### Reactive Oxygen Species Analysis

ROS were measured using the fluorescent dye, 2′,7′‐dichlorofluorescein diacetate (DCFDA) (Thermo Fisher Scientific) in live cell samples and in solution alone. In solution, 4.9 mg of 1 mM DCFDA was prepared with 10 mL of 100% ethanol. DCFDA was deacetylated to the 2′,7′‐dichlorofluorescein form by adding 500 μL of DCFDA solution to 2 mL of 0.01 m sodium hydroxide (Thermo Fisher Scientific) and left in the dark at 4 °C for 30 min. An amount of 10 mL of 25 mM sodium dihydrogen orthophosphate (Sigma‐Aldrich) was then added to the solution to neutralize the reaction. An amount of 4.9 mg of 1 mM DCFDA was prepared with 10 mL of dimethyl sulfoxide (Sigma‐Aldrich).

Cells were plated in a 48‐well plate at a concentration of ≈1 × 10^4^ cells using RPMI‐1640 media not containing phenol red and left to attach overnight. The next morning, cells were washed with PBS and 100 μL NP solutions at an approximate gold concentration of 0.199 μmol mL^−1^ of AuT and 0.083 μmol mL^−1^ of AuSiO_2_ NPs were added to wells containing cells and for measurement in solution and incubated for 2 h. After 2 h, wells containing cells were washed with PBS. 30 μL of 10 μM DCFH was added to 100 μL of NPs alone in solution. An amount of 200 μL of 1 mg mL^−1^ horseradish peroxidase (Sigma‐Aldrich) diluted in PBS was used to catalyze the reaction. In wells containing cells, 100 μL of PBS was added to the wells and 30 μL of 10 μM DCFDA was added. Negative and positive (using hydrogen peroxide [Sigma‐Aldrich]) controls were implemented.

Under protection from light, well plates were transported to the Lyell McEwin Hospital for an X‐ray irradiation as described previously in “X‐ray irradiation.” One hour postirradiation, samples were analyzed using a FLUOstar Optima Plate Reader at fluorescence excitation of 495 nm and emission of 525 nm.

##### Statistical Analysis

Results from the DNA damage repair marker, *γ*H2AX, are presented in box and whisker plots. The boxes incorporate 25–75 percentiles (interquartile range) of the dataset, separated by the red horizontal line representing the median. The whiskers represent 1.5 × the interquartile range, and red plus signs indicate outlier data beyond this range. Green diamonds represent means.

On each dataset, MATLAB software was used to conduct one‐way ANOVA significance tests to determine significance in the means between cell lines and NPs. A blue line indicates a statistical significance between the means (*p* < 0.05). A purple line indicates a high statistical significance between the means (*p* < 0.001). A red line indicates a very high statistical significance between the means (*p* < 0.0001). A figure with black lines indicates that there is no statistical significance between variables, but every other comparison is statistically significant (*p* < 0.05).

## Conflict of Interest

The authors declare no conflict of interest.

## Supporting information

Supplementary Material

## Data Availability

The data that support the findings of this study are available from the corresponding author upon reasonable request.
