# Supplementary material for: Quantitative Single‐Cell Comparison of Sensitization to Radiation and a Radiomimetic Drug for Diverse Gold Nanoparticle Coatings
Source: Small Sci. 2024 Jun 16;4(9):2400053. doi: 10.1002/smsc.202400053 (PMC11935229; doi:10.1002/smsc.202400053)
Supplement: Supplementary file 1 — Supplementary Material [file SMSC-4-2400053-s001.pdf]

Supporting Information

Quantitative single-cell comparison of sensitization to radiation and a radiomimetic drug for diverse gold nanoparticle coatings

Douglas Howard[1,2], Tyron Turnbull[1], Puthenparampil Wilson[3,4], David Paterson[5], Valentina Milanova[1], Benjamin Thierry[1], Ivan Kempson[1*]

[1]Future Industries Institute, University of South Australia, Mawson Lakes, South Australia 5095, Australia

[2]Department of Nuclear Medicine, University Hospital Essen, Hufelandstrasse 55 45122 Essen, Germany

[3]UniSA STEM, University of South Australia, Mawson Lakes, South Australia 5095, Australia

[4]Department of Radiation Oncology, Royal Adelaide Hospital, Adelaide, South Australia 5000, Australia

[5]Australian Synchrotron, ANSTO, 800 Blackburn Road, Clayton, Victoria 3168, Australia

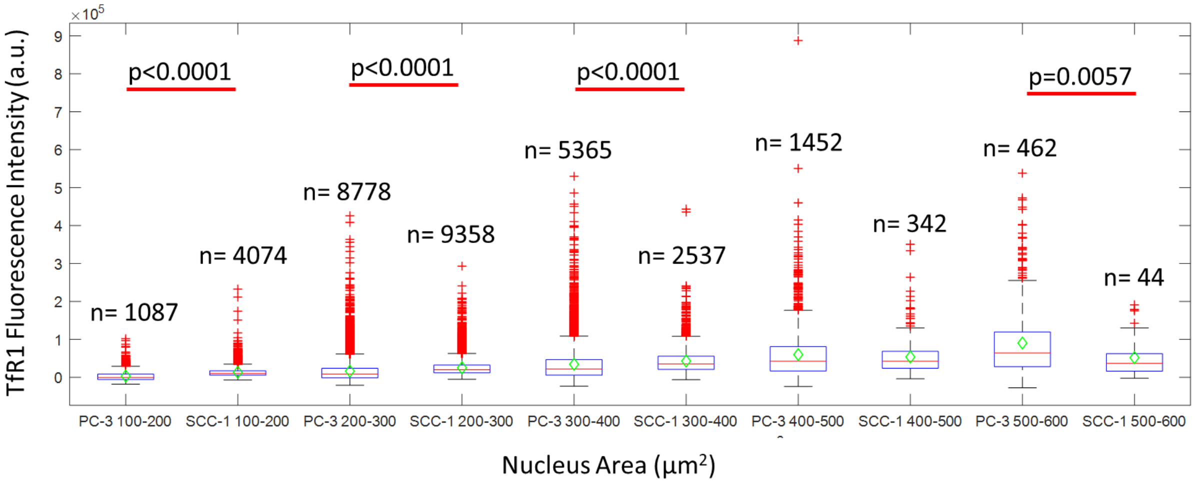

Figure S1. Cluster of Differentiation 71 (CD71), also known as Transferrin receptor protein 1 (TfR1), expression comparing PC 3 and SCC1 cells for increasing nucleus size ranges. For smaller cells, SCC-1 cells exhibit greater expression that PC-3 cells. However for larger cells the PC-3 cells have greater expression than the SCC-1 cells.

Table S1. NP association statistics for the AuPEG, AuT and $AuSiO_2$ NPs in PC-3 and SCC-1 cells; including the cell count, mean, standard deviation, standard error in the mean, and the range.

	NP Condition	n	Mean Au mass (pg)	Std Dev (pg)	Std Error (pg)	Range (pg)
PC-3	AuPEG	198	4.47	6.05	0.43	0.003-56.5
	AuT	646	12.5	14.7	0.58	0.014-149
	$AuSiO_2$	506	0.73	0.82	0.036	0.0001-4.4
SCC-1	AuPEG	306	4.57	5.63	0.32	0.003-49.6
	AuT	402	17.3	13.7	0.68	0.026-95.8
	$AuSiO_2$	203	1.85	2.58	0.18	0.006-34.1

Table S2. Summary of the γH2AX foci per cell in the PC-3 and SCC-1 cell lines for no NPs (control), AuPEG, AuT and $AuSiO_2$ NP conditions for 0Gy and 4Gy irradiation.

	Irradiation Condition	NP condition	n	Mean (foci per cell)	St. dev (foci per cell)	Median (foci per cell)
PC-3	0Gy	Control	993	4.3	12.5	0
		AuPEG	198	1.7	6.3	0
		AuT	646	4.4	11.4	0
		$AuSiO_2$	506	1.8	8.8	0
	4Gy	Control	463	39.6	30.5	36
		AuPEG	418	31.1	27.1	25
		AuT	767	50.3	32.6	49
		$AuSiO_2$	665	30.4	28.7	22
SCC-1	0Gy	Control	698	1.9	9.0	0
		AuPEG	496	1.6	8.9	0
		AuT	640	3.4	14.0	0
		$AuSiO_2$	539	1.3	9.4	0
	4Gy	Control	1013	21.7	25.9	12
		AuPEG	696	28.1	27.6	21
		AuT	1087	27.9	27.4	20
		$AuSiO_2$	921	30.2	28.5	25

Table S3. Summary of the γH2AX foci per cell in the PC-3 cell line irradiated with a 4 Gy X-ray irradiation and sorted by the amount of gold associated with individual cells for the AuPEG, AuT and $AuSiO_2$ NP conditions.

NP condition	Gold associated (pg)	n	Mean (foci per cell)	St. dev (foci per cell)	Median (foci per cell)
AuPEG	0-1	88	28.9	29.2	21
	1-3	122	32.3	27.0	27.5
	3-5	68	29.0	26.7	22
	5-7	46	36.8	27.6	30
	7-10	36	22.9	17.5	21
	10-15	36	32.4	26.6	25.5
	15-20	11	33.0	26.7	22
	>20	11	44.9	33.9	39
AuT	0-1	85	37.7	36.1	29
	1-3	102	46.7	33.4	46.5
	3-5	77	48.8	30.2	48
	5-7	67	46.2	32.0	47
	7-10	91	48.7	29.2	47
	10-15	144	57.2	29.7	55
	15-20	79	58.6	33.8	61
	>20	122	52.9	33.3	48
$AuSiO_2$	0-1	420	28.2	27.1	19.5
	1-3	115	33.0	30.2	25
	3-5	51	32.1	27.8	24
	5-7	24	39.3	30.4	36
	7-10	26	43.5	40.4	30.5
	10-15	19	26.6	25.4	22
	15-20	5	50.4	47.0	35
	>20	5	20	21.0	12

Table S4. Summary of the γH2AX foci per cell in the SCC-1 cell line irradiated with a 4 Gy X-ray irradiation and sorted by the amount of gold association with individual cells for the AuPEG, AuT and $AuSiO_2$ NP conditions.

NP condition	Gold association (pg)	n	Mean (foci per cell)	St. dev (foci per cell)	Median (foci per cell)
AuPEG	0-1	42	34.7	30.5	31.5
	1-3	159	35.8	30.8	30
	3-5	128	29.6	26.5	25
	5-7	127	25.9	25.2	22
	7-10	115	26.0	26.7	18
	10-15	80	17.0	18.3	11.5
	15-20	30	18.4	30.6	3.5
	>20	15	28.4	30.1	21
AuT	0-1	74	31.7	33.0	20
	1-3	113	27.0	25.7	19
	3-5	128	32.7	29.3	31.5
	5-7	117	24.2	25.7	16
	7-10	158	30.6	27.3	24
	10-15	206	27.0	24.7	21.5
	15-20	130	25.1	26.5	15
	>20	159	26.1	29.1	16
$AuSiO_2$	0-1	149	41.8	32.1	41
	1-3	42	20.6	22.5	11
	3-5	-	-	-	-
	5-7	1	74.0	0	74
	7-10	1	67.0	0	67
	10-15	-	-	-	-
	15-20	-	-	-	-
	>20	-	-	-	-

Table S5. Summary of the γH2AX foci per cell in the PC-3 and SCC-1 cell lines after exposure with the radiomimetic drug, Neocarzinostatin (NCS) for a control, AuPEG, AuT and $AuSiO_2$ NP conditions.

	Irradiation Condition	NP Condition	n	Mean (foci per cell)	St. dev (foci per cell)	Median (foci per cell)
PC-3	No NCS	Control	263	2.36	10.2	0
		AuPEG	186	1.71	9.27	0
		AuT	280	1.36	9.45	0
		$AuSiO_2$	382	0.40	3.42	0
	NCS	Control	514	25.6	32.4	8
		AuPEG	314	21.4	30.9	3.5
		AuT	669	21.6	30.0	4
		$AuSiO_2$	261	28.1	32.5	14
SCC-1	No NCS	Control	781	0.35	2.91	0
		AuPEG	385	0.69	4.89	0
		AuT	651	1.05	5.93	0
		$AuSiO_2$	402	0.93	5.59	0
	NCS	Control	158	44.3	30.7	41
		AuPEG	270	36.1	29.1	33
		AuT	246	36.9	25.7	33
		$AuSiO_2$	538	29.8	23.5	28

A

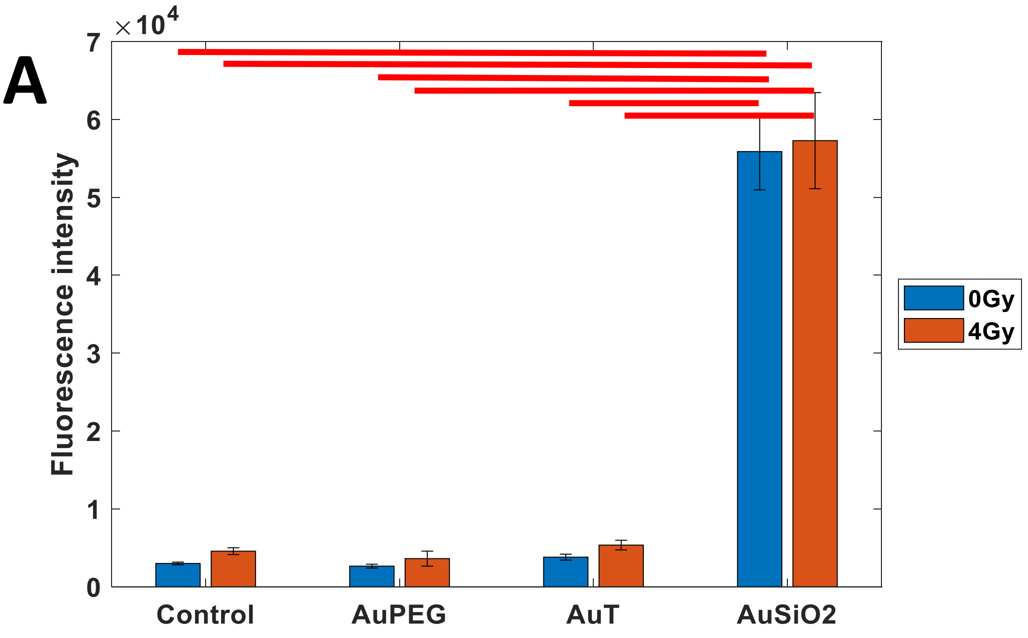

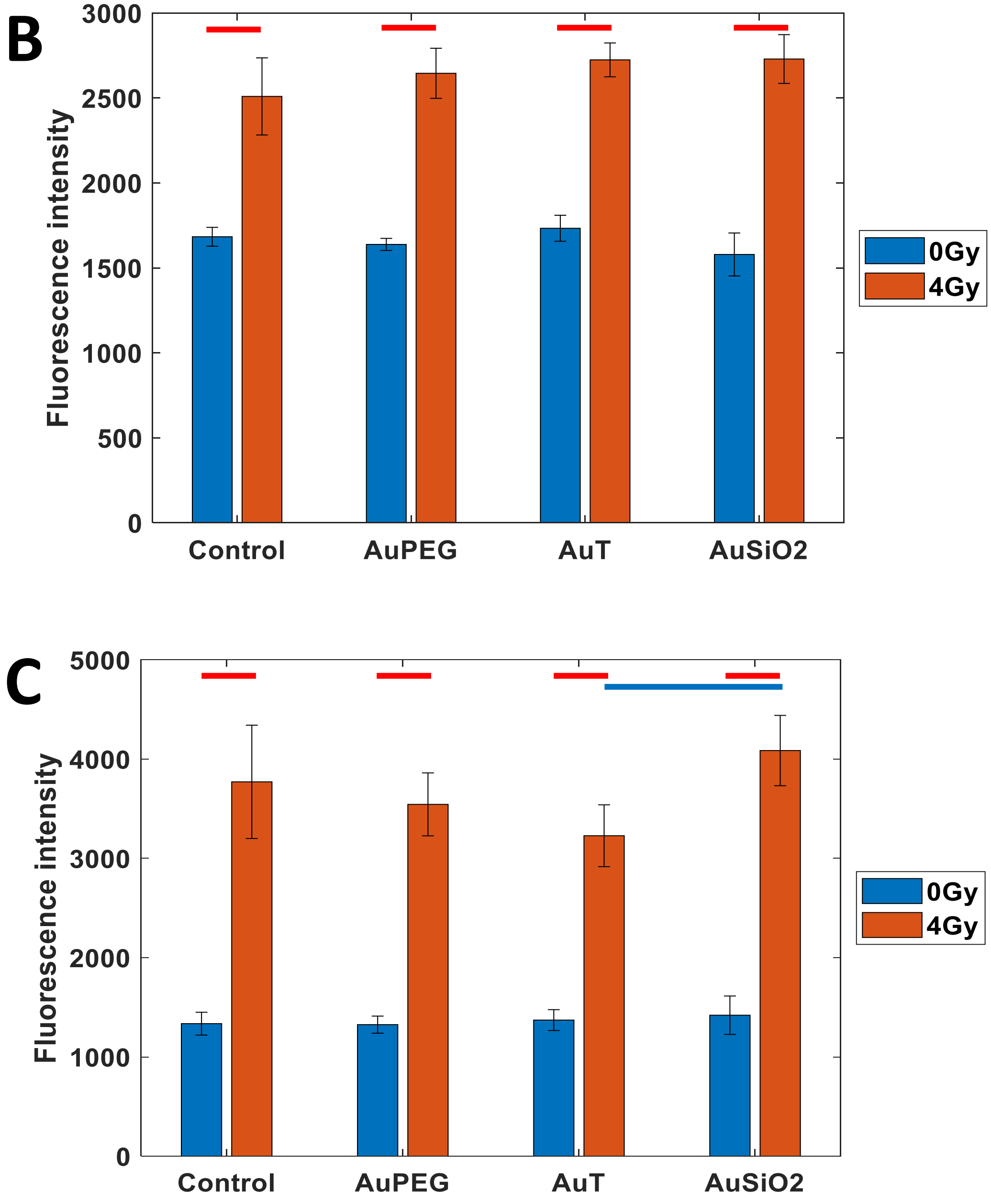

Figure S2. Reactive oxygen species generation measured by the fluorescence intensity of the dye, DCFDA, with AuPEG, AuT and AuSiO2 NP conditions without cells (A), and in PC-3 (B) and SCC-1 (C) cell lines after a 4Gy X-ray dose irradiation. A one-way ANOVA significance test has been carried out on each dataset to determine statistical significance between the means. Red lines indicate a statistical significance between the means ($p<0.0001$) and blue lines indicate a statistical difference between the means ($p<0.05$).

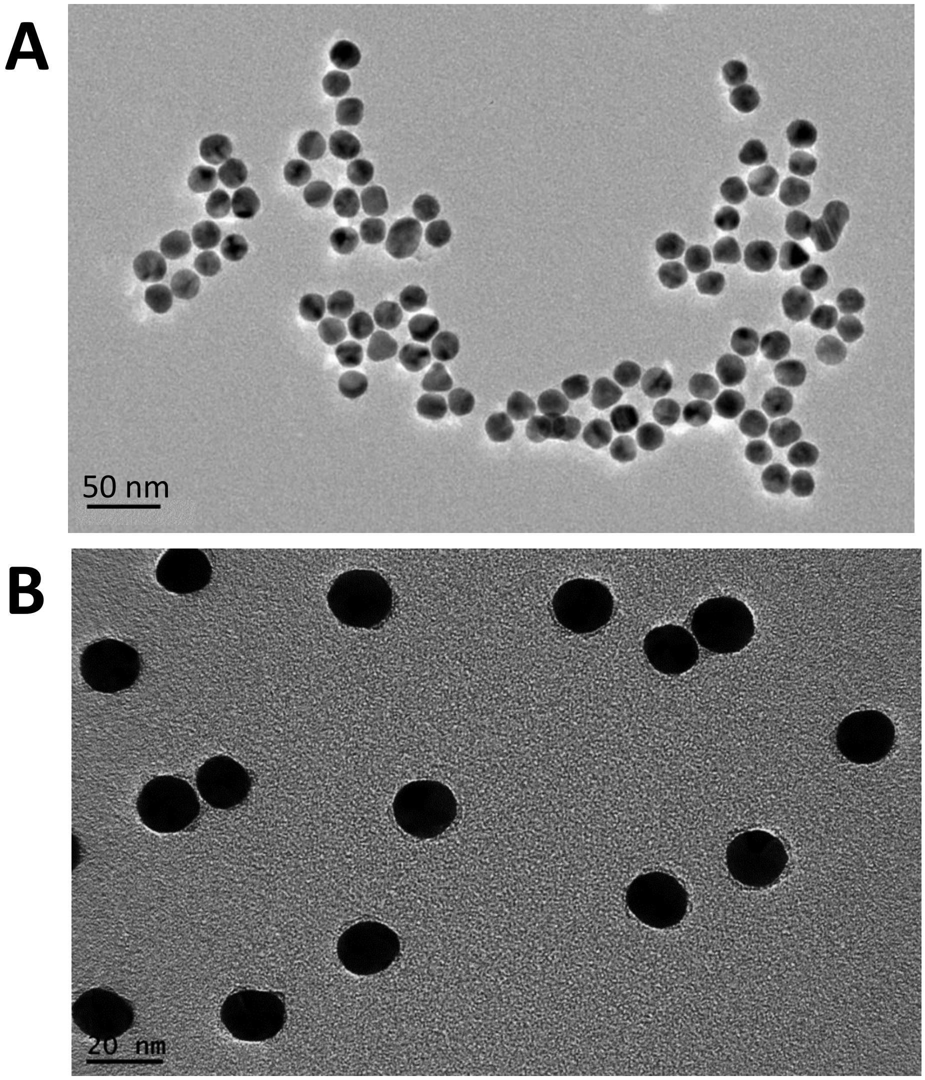

Figure S3. Transmission electron microscopy image of gold core (A) and gold core/silica shell NP, $AuSiO_2$ (B). Contrast has been increased to show the thin silica shell.
